# Subhepatic appendicitis in a 27-year-old male: a case report from Odahulle General Hospital of Ethiopia

**DOI:** 10.3389/fgstr.2024.1503818

**Published:** 2025-01-14

**Authors:** Abduletif Haji Ababor Abagojam, Remedan Jemal Dekema, Tamirat Godebo Woyimo, Jafer Yasin Mohammed, Kedir Negesso Tukeni

**Affiliations:** ^1^ Department of Surgery, Jimma University, Jimma, Ethiopia; ^2^ Department of Surgery, Odahulle General Hospital, Jimma, Ethiopia; ^3^ Department of Internal Medicine, Jimma University, Jimma, Ethiopia

**Keywords:** subhepatic appendix, atypical acute appendicitis, acute abdomen, appendectomy, surgery, appendix location, Odahulle General Hospital, Ethiopia

## Abstract

The appendix is a small, tube-shaped organ that connects to the cecum, the beginning of the large intestine. Though its role is unknown, it can become infected, resulting in acute appendicitis, which, if not detected and treated early, can lead to serious consequences. Though the symptoms and signs of acute appendicitis are straightforward in most cases, atypical locations might result in unexpected presentations, which can lead to complications as it might not be detected and treated early. This case report describes a 27-year-old Black Ethiopian male patient who presented with right upper abdominal pain, low-grade fever, palpitations, and diarrhea. He also had some episodes of vomiting of ingested matter. Upon physical examination, the patient appeared acutely sick with some degree of tachycardia. An abdominal examination revealed right upper abdominal quadrant tenderness, though there was no palpable mass noted. Laboratory investigations were unremarkable apart from the stool examinations which revealed many pus and red blood cells and was full of actively motile bacteria. An abdominal ultrasound showed an enlarged subhepatic appendix with an internal fecalith 1 cm in depth, that was partially compressible, with no obvious peri appendiceal free fluid or other pathology. An assessment of subhepatic acute appendicitis was suspected and an emergency operation was conducted which revealed an inflamed subhepatic appendix that was on the verge of rupture. At a subsequent follow-up, the patient had significantly improved as evidenced by the lack of symptoms including abdominal pain, fever, and palpitation. Subhepatic appendix is a rare condition caused by either the non-descent of the cecum or intestinal malrotation during early development. As its presentation is not classical and hence mimics other pathologies, the diagnosis may be overlooked, resulting in perforation and abscess formation and leading to increased morbidity and possibly mortality. A high level of suspicion is required for early diagnosis and treatment to improve patient outcomes.

## Highlights

- Diagnosing subhepatic appendicitis requires a high level of suspicion as it might mimic other illnesses, including biliary pathology.
- The acute nature of this disease, along with the significant risk of perforation and abscess as it might be missed early, make surgical intervention difficult.
- Laparoscopy is a valuable tool in cases of atypical abdominal pain both for diagnosis and treatment.

## Introduction

The appendix is a small, tube-shaped organ that connects to the cecum, the beginning of the large intestine. Though its role is unknown, it can become infected, resulting in acute appendicitis, which, if not detected and treated early, can lead to serious consequences. Appendicitis is one of the acute abdomen conditions requiring urgent surgical intervention. Normal appendiceal structure and classical appearance are well known, although there are abnormalities, as documented in the literature (1–6). A high level of suspicion and awareness of these anatomical variants is required to effectively diagnose and manage appendicitis. The incidence of a subhepatic appendix, one of the anatomical variants, was reported to be 0.08% (1), with the first case of subhepatic appendicitis caused by a cecum that did not descend (2). Most of the anatomical abnormalities have been linked to intestinal malrotation rather than a lack of cecal descent (5, 6). As subhepatic appendicitis does not manifest in the traditional manner, it is easy to mistake it for other diseases, including biliary pathology. Furthermore, subhepatic appendicitis appears to be more common among the elderly, contributing further to the lack of a timely diagnosis (7). In many cases, it has a chronic course with ill-defined right flank and right upper quadrant abdominal pain, as was the case in this report, and the diagnosis is frequently made via laparoscopy. Perforation and abscess formation are important complications related to the late diagnosis of acute appendicitis (5, 6).

Symptoms of acute appendicitis typically begin with pain in the center of the abdomen periumbilically, which then spreads to the right lower abdomen or into where the appendix could be located, within a few hours, which worsens over time. The pain may even worsen with movement, coughing, or pressing on the area (8).

Presentations can be atypical in some cases; the pain can be less severe, develop slowly, or occur in a different location. This is more likely to occur in pregnant women, small children, and the elderly. Others might simply feel or be unwell, have a loss of appetite, have a high fever, have constipation or diarrhea, and experience sudden confusion (especially in the elderly). If appendicitis is not treated promptly due to an atypical presentation as a result of location, as in this case report, it could rupture, leading to generalized peritonitis and then to features of sepsis that could lead to prolonged morbidity and even mortality (7, 8). Therefore, though the symptoms and signs of acute appendicitis are straightforward in most of the cases, there might be atypical presentation due to which diagnosis might be missed or at least delayed. As a result, there are active guidelines that help with the diagnosis and direct treatment that include the use of clinical scores and imaging, indications and timing of surgery, use of non-operative management and antibiotics, laparoscopy and surgical techniques, intra-operative scoring, and peri-operative antibiotic therapy (8).

## Case presentation

A 27-year-old Black Ethiopian male from a rural part of the Oromia region who farms to earn a living, and with no known chronic medical condition, presented with a 5-day history of periumbilical abdominal pain which later radiated to the right upper abdominal area, a low-grade fever, palpitations, and diarrhea. He also had some episodes of vomiting of ingested matter. Systemic inquiries were unremarkable. He denied having previous similar complaints or having a family history of similar illnesses. He was not on any medications, did not use alcohol or recreational drugs, and had no history of smoking. He also had no personal or family history of diabetes, hypertension, cardiovascular disease, or any other recognized medical conditions. He usually ate enjera with wot, bread, and, intermittently, meat, which had not changed recently.

Upon physical examination, he appeared acutely ill, with a blood pressure of 100/60 mmHg, pulse rate of 104 beats per minute, respiration rate of 22 breaths per minute, body temperature of 38.2°C, and oxygen saturation of 98% at room air. He was a slim male who weighed 59 kg, was 169 cm tall, and had a calculated BMI of 20.70 kg/m2. There were pink conjunctivae but there was no jaundice or cyanosis. There was right upper abdominal quadrant tenderness, though no mass was found (**Table 1**).

**Table 1:** Summary of investigations performed during the patient’s hospitalization.

Variable	Results on the day of admission	Reference ranges
Hematocrit (%)	36.4	36.0–46.0
Hemoglobin (mg/dl)	15.9	11—16.5
White cell count (per µl)	6080	4000—15000
Platelet (per µl)	262,000	150,000—450000
Mean corpuscular volume (fl)	88.5	82-100
Red−cell distribution width (%)	15.8	11.5–14.5
ESR (mm/hr.)	32	0-20
Creatinine (mg/dl)	1	0-5—1.2
Blood urea nitrogen (mg/dl)	41	7—20
Sodium (mmol/L)	139	135—145
Potassium (mmol/L)	3.48	3.5—5.5
Chloride (mmol/L)	99.3	98—107
Calcium (mg/dl)	9.401	8.5–10.5
AST (IU/L)	35	0—40
ALT (IU/L)	29	0—41
ALP (IU/L)	195	40—130
LDH(U/L)	120	140-280
Serum Bilirubin (mg/dl) Total	1.1	0.3-1.2
Serum Bilirubin (mg/dl) direct	0.5	0.0-0.2
Reticulocyte count (%)	2.2	0.7–2.5
Prothrombin time (sec)	10.1	10—14
APTT (sec)	29.2	22—38
INR	0.84	0.7—1.2
D-dimer	<0.5	<0.5
Fibrinogen(mg/dL)	112	100-200
Hs-CRP (mg/L)	1	0-1
Procalcitonin(ng/mL)	1.51	< 0.1
HIV test	Non-reactive	Non-reactive
Vit-B12(pg/ml)	512	
Serum folate(ng/ml)	>20	
Stool examinations	Few pus cells	Negative
Serum VDRL test	Non-reactive	Non-reactive
Hepatitis B surface antigen	Negative	Negative
Hepatitis C antibody test	Negative	Negative
Urinalysis	Unremarkable	
Stool *H Pylori* test	Negative	
Serum Albumin (g/dl)	3.8	3.3–5.0

ALP, Alkaline Phosphatase; ANA, antinuclear antibody; APTT, Activated partial thromboplastin time; AST/ALT, Aspartate Transaminase/Alanine Transaminase; ESR, Erythrocyte sedimentation rate; HIV, Human Immunodeficiency Virus; VDRL, Venereal Disease Research Laboratory.

Further investigation with abdominal ultrasonography revealed an enlarged subhepatic appendix measuring 6.55mm in diameter with an internal fecalith 1 cm in depth, that was partially compressible. There was no peri appendiceal free fluid collection and no intraabdominal mass or lymphadenopathy was noted. The patient’s erythrocyte sedimentation rate and procalcitonin was increased to 32mm/hr and 1.51ng/mL, respectively.

**Figure 1:**
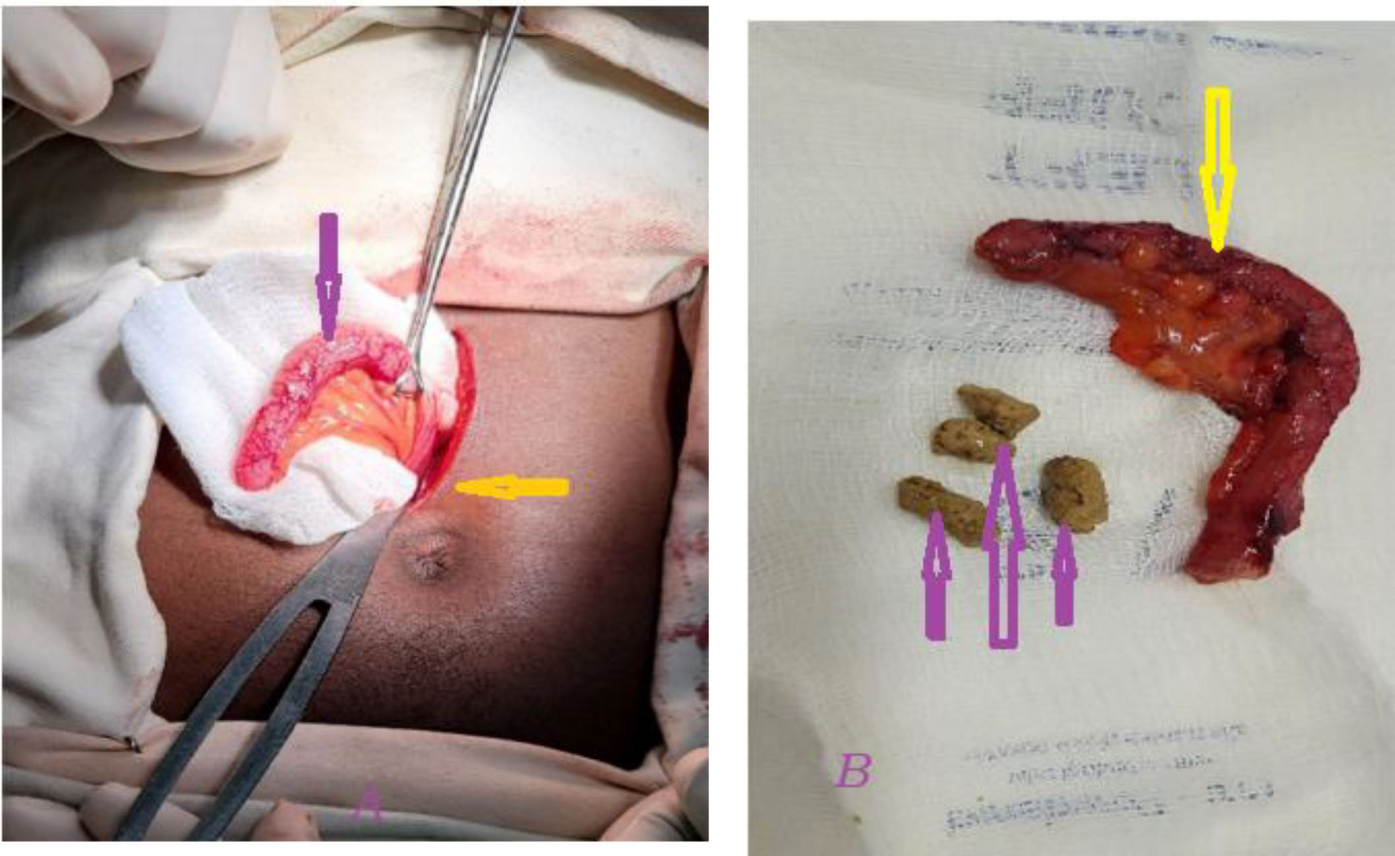
There was an enlarged and inflamed subhepatic appendix. **(A)** the yellow arrow shows the level of surgical incision at the level of the umbilicus while the pink arrow shows the removed appendiceal tissue with signs of inflammation. **(B)** An inflamed appendix measuring 6.55mm in diameter (yellow arrow) with an internal fecalith 1 cm in depth (pink arrow), suggestive of acute appendicitis.

With the diagnosis of subhepatic acute appendicitis, the patient was taken to the operation theatre after securing intravenous lines for resuscitation and antibiotics as per recommendations, and an appendectomy was conducted after stabilizing his hemodynamic status. The intraoperation findings revealed an inflamed subhepatic appendix which was on the verge of rupture (**Figure 1**). The patient was discharged after 48 hours as he had no fever, abdominal pain, vomiting, or diarrhea, and his bowel was confirmed to be active as he passed feces and flatus.

At a subsequent follow-up, the patient was significantly improved as evidenced by the lack of symptoms including abdominal pain, fever, or palpitation, and a clean surgical wound.

## Discussion

The incidence of subhepatic appendix is rare and has been found in 0.08% of patients diagnosed with appendicitis (1). It is thought to be due to either a cecum that does not descend or intestinal malrotation during embryonic development (2, 5, 6). Though appendicitis is a common acute surgical condition with a well-documented classical presentation with normal anatomy, there are aberrations (1–6) that mimic other diseases; therefore, a high index of suspicion and awareness of these anatomical variants is required to correctly diagnose and safely manage appendicitis.

As subhepatic appendicitis does not show in the usual way, it is easy to confuse with other conditions, including biliary pathology. Furthermore, subhepatic appendicitis appears to be more common among the elderly, which adds to the uncertainty surrounding the diagnosis (7). In many situations, it is a subacute condition with ill-defined right flank and right upper quadrant pain, further complicating an early diagnosis.

Subhepatic cecum occurs due to incomplete rotation and fixation/maldescent of the fetal foregut (5, 9, 10). Differential diagnoses include acute cholecystitis, liver abscess, perforated duodenal ulcer, and right renal calculus, which necessitates imaging, including abdominal ultrasound, that reveals the pathology in each of the scenarios, as was used in this case report. Although an abdominal ultrasound is the most commonly used diagnostic technique, as in our case, computed tomography (CT) scans also provide a high sensitivity for diagnosis (5). In many situations, a diagnosis is made via laparoscopy, particularly when a CT scan is ambiguous (10). A CT scan can reveal that these patients have subhepatic appendicitis and better differentiate it from alternative diagnoses (5).

Patient presentation with physical findings is usually an important initial step in the diagnosis of acute appendicitis although clinical presentation can be atypical in those with abnormal anatomy, as documented in this case report. Blood tests are usually normal with mild leukocytosis (7, 8). An ultrasound scan revealed a tubular hypoechoic formation in the location that corresponded to the area of greatest tenderness. The surgery that was conducted showed an inflamed appendix in a subhepatic location in this case report.

## Conclusion

A subhepatic appendix is an uncommon condition caused by either the non-descent of the cecum or intestinal malrotation during early development. When inflamed, this can lead to misdiagnosis as it mimics other pathologies in organs typically found there, resulting in perforation and abscess formation, which can increase morbidity and possibly mortality. A high level of suspicion is essential for an early diagnosis and intervention in order to improve patient outcomes.

## Patient perspective

I’ve been suffering with agonizing pain in my right upper abdomen for the past five days, along with nausea and a low fever. Though I am feeling better after the surgery, I am still having mild pain at the wound site. As a result, I beg that doctors assist me in preventing any possible complications.

## Data Availability

The original contributions presented in the study are included in the article/supplementary material. Further inquiries can be directed to the corresponding author.
